# CXCR6 Expression Is Important for Retention and Circulation of ILC Precursors

**DOI:** 10.1155/2015/368427

**Published:** 2015-10-01

**Authors:** Sylvestre Chea, Cécilie Possot, Thibaut Perchet, Maxime Petit, Ana Cumano, Rachel Golub

**Affiliations:** ^1^Unité de Lymphopoièse, Département d'Immunologie, Institut Pasteur, 75015 Paris, France; ^2^Université Paris Diderot, Sorbonne Paris Cité, Cellule Pasteur, Paris, France; ^3^Inserm U668, Paris, France

## Abstract

Innate lymphoid cells are present at mucosal sites and represent the first immune barrier against infections, but what contributes to their circulation and homing is still unclear. Using *Rag2* ^−/−^ *Cxcr6* ^*Gfp*/+^ reporter mice, we assessed the expression and role of CXCR6 in the circulation of ILC precursors and their progeny. We identify CXCR6 expressing ILC precursors in the bone marrow and characterize their significant increase in CXCR6-deficient mice at steady state, indicating their partial retention in the bone marrow after CXCR6 ablation. Circulation was also impaired during embryonic life as fetal liver from CXCR6-deficient embryos displayed decreased numbers of ILC3 precursors. When injected, fetal CXCR6-deficient ILC3 precursors also fail to home and reconstitute ILC compartments *in vivo*. We show that adult intestinal ILC subsets have heterogeneous expression pattern of CXCR6, integrin *α*_4_ *β*_7_, CD62L, CD69, and CD44, with ILC1 and ILC3 being more likely tissue resident lymphocytes. Intestinal ILC subsets were unchanged in percentages and numbers in both mice. We demonstrate that the ILC frequency is maintained due to a significant increase of ILC peripheral proliferation, as well as an increased proliferation of the *in situ* ILC precursors to compensate their retention in the bone marrow.

## 1. Introduction

The family of innate lymphoid cells (ILCs) concerns cells devoid of rearranged antigen receptors. It comprises conventional EOMES expressing NK (cNK) cells and the helper subsets composed of three groups (ILC1, ILC2, and ILC3) in analogy with the T-helper cell nomenclature [1]. ILC1 designates the group of T-bet expressing cells that are producing the IFN*γ* whereas ILC2 includes ROR*α*/GATA3 dependent cells producing type Th2 cytokines. ILC3 includes different subsets that are all ROR*γ*t dependent and produce IL-17 and/or IL-22 (for review, see [2]). In the intestine, the ILC3 populations constantly interact with the microbiota, dietary compounds, epithelial factors, and cytokines to preserve the epithelial barrier integrity. IL-22 producing cells from the ILC3 group mainly concerns the subset that expresses the NK cell receptor NKp46 (termed NCR^+^ ILC3). In mice, this intestinal subset has been shown to protect against infection with the pathogen* Citrobacter rodentium* [3–5]. NCR^+^ ILC3 cells are CCR6^−^ and do not produce IL-17A. They are rare in cryptopatches compared to CD4^+^ ILC3 (LTi–like) cells that express CCR6 and produce IL-17A. This LTi–like subset is the adult counterpart of the LTi cells present during fetal life and is crucial for the development of lymph nodes (LN) and Peyer's patches. A third subset of double negative (CD4^−^ NKp46^−^) ILC3 is also present in the lamina propria. It is usually denominated by NCR^−^ILC3 [6–8]. Intestinal ILC2 populations are crucial against parasitic infections [9–11] and may also be implicated in the regulation of gut homeostasis by limiting the inflammation [12].

The migration of lymphocytes to specific tissues is driven by the upregulation of adhesion and chemokine receptors. The T lymphocytes are imprinted with specific trafficking programs that are currently well known [13]. However, little is known concerning the circulation of ILC and their progenitors. All ILC groups derive from a bone marrow (BM) precursor that expresses the ID2 transcription factor and the common *γ* chain of the interleukin 2 receptor [2]. Initial steps of ILC development start in the bone marrow. Intestinal lamina propria and spleen could also support the late stages of ILC differentiation, especially for the ILC3 lineage since precursors are unable to upregulate ROR*γ*t in the bone marrow [14]. Using mice bearing a targeted insertion of a GFP-reporter into the* Cxcr6* locus (*Cxcr6* ^*Gfp/+*^ mice) [15], we have previously shown that a subset of ILC precursors both in the adult bone marrow and fetal liver expresses CXCR6 [14]. CXCR6 has also been described as expressed by most of the ILC intestinal subsets [16]. Homing and trafficking are achieved by a specific combination of adhesion molecules and chemokine receptors, and CXCR6 expression could be critical and correlated with differential homing and/or function of ILC subsets. It has been shown, for example, that hepatic NK cells rely on CXCR6 expression for the persistence of memory NK cells [17]. Hence, we decided to evaluate the role of CXCR6 in the circulation of both ILC progenitors and ILC subsets.

In this study, we assess the effect of CXCR6 deficiency on ILC precursors and ILC subsets from the bone marrow, intestinal lamina propria (LP), and mesenteric lymph nodes (mLN). The effect of CXCR6 loss could be analyzed using the* Rag2* ^−*/*−^ *Cxcr6* ^*Gfp/Gfp*^ mouse model since the GFP reporter insertion simultaneously inactivates the corresponding* Cxcr6* allele [15], and* Rag2* deletion ensures a proper ILC analysis devoid of T-cell contamination. We have observed an increased percentage of bone marrow CXCR6^+^ ILC precursors without any concomitant proliferation of those precursors, nor loss of differentiation potential, which suggests active bone marrow retention of ILC precursors after the CXCR6 loss. Similarly, CXCR6 contributes to circulation of fetal ILC3 precursors in the fetal liver (FL) as CXCR6-deficient fetal livers were partially depleted of ILC3 precursors. Using injection and reconstitution analysis, we show that CXCR6 is required for circulation of ILC3 precursors to intestinal LP or liver.

We show that pattern expression of CXCR6 and integrin *α*_4_ *β*_7_ and egress or circulation markers such as CD62L, CD69, and CD44 among ILC are heterogeneous and only intestinal ILC1 and ILC3 subsets were more likely to be tissue resident ILC populations. Finally, we show that CXCR6-deficiency does not alter the homeostatic balance of intestinal ILC subsets. However, those subsets were seeded and enriched in dividing and recently divided cells when CXCR6 deficiency occurs. The bone marrow derived ILC progenitor was similarly highly active and proliferative in CXCR6-deficient intestinal lamina propria, showing that this high proliferative state compensates retention of the bone marrow precursors.

## 2. Materials and Methods

### 2.1. Mice

*Rag2* ^−*/*−^ *Cxcr6* ^+/+^,* Rag2* ^−/−^ *Cxcr6* ^*Gfp*/+^,* Rag2* ^−/−^ *Cxcr6* ^*Gfp/Gfp*^,* Cxcr6* ^*Gfp/*+^ *Id2* ^*Yfp/*+^, and Ly5.1* Rag2* ^−/−^ *γc* ^−*/*−^ mice were bred in the animal facilities at Pasteur Institute, Paris. Mice were cared for in accordance with Pasteur Institute guidelines in compliance with European animal welfare regulations, and all animal studies were approved by Pasteur Institute Safety Committee in accordance with French and European guidelines.

### 2.2. Cell Preparation

Bone marrows (BM) were flushed out of femurs and tibias; mesenteric lymph nodes (mLN), fetal livers (FL), and fetal spleens (FS) were mechanically dissociated to obtain cell suspensions. Additionally, BM red blood cells were lysed by incubation with ammonium chloride-potassium bicarbonate solution. Cells from BM, mLN, and FL were magnetically depleted of Lin^+^ cells via staining with biotin labeled antibodies to lineage markers, followed by the use of Streptavidin MicroBeads (Miltenyi).

Small intestine was washed from its contents by PBS injection, and Peyer's patches, if present, were removed. After being cut open longitudinally, small intestine was cut in 1 cm fragments. Fragments of small intestine were incubated 30 min at 37° and 5% CO_2_ in RPMI medium (Gibco) plus 20% fetal calf serum (FCS), HEPES buffer (10 *µ*M, Sigma-Aldrich), and then vortexed thoroughly for 4 minutes for removal of epithelial cells and intraepithelial lymphocytes. Remaining fragments of small intestine were incubated 30 min at 37° and 5% CO_2_ in RPMI medium plus 20% FCS, HEPES Buffer (10 *µ*M), and collagenase type VIII (250 *µ*g/mL, Sigma) for the isolation of LP lymphocytes. Cell suspension was centrifuged, resuspended in a 40% solution of Percoll (GE Healthcare), and underlaid with a 75% solution of Percoll. After centrifugation 20 min at 600 g, cells were collected at the 40–75% interface.

All cells were then collected in cold HBSS (Gibco) plus 1% FCS. Cell suspensions were counted on Malassez cell, and dead cells were excluded using Trypan Blue.

### 2.3. Flow Cytometry

After antibody staining, cells were washed and dead cells were excluded by Propidium Iodide staining (250 ng/mL, Sigma-Aldrich). Cells were stained intracellularly using Foxp3 Permeabilzation/Fixation Kit according to manufacturers notice (eBiosciences). Cells were then incubated for 5 min with DAPI (4,6-diamidino-2-phenylindole, 2 *µ*g/mL, Life Technologies), and washed prior to cell acquisition. FACSCanto II and LSR Fortessa (BD Biosciences) were used for flow cytometry acquisition, with Diva6 software (BD Biosciences), and analyzed with FlowJo v8 software (TreeStar). For visual purposes, only 8000 events maximum are shown in each FACS dot plot.

Cells were purified with a FACSAria III sorter (BD Biosciences). Cells were recovered in PBS for cell injection, or OPTIMEM (Gibco) plus 10% FCS for cell culture.

### 2.4. Antibodies

Streptavidin was coupled to PECy5. The following antibodies were either biotinylated or coupled to fluorochromes (Alexa488, PerCPCy5.5, PE, PECy7, APC, Alexa 660, APCCy7, BV421, eFluor 450, HZ V500, BV605, BV650, BV711, BV786): Ly76 (TER-119), Gr-1 (RB6-8C5), CD11c (HL3), CD3*ε* (145-2C11), CD19 (6D5), CD8*α* (53-6.7), CD5 (53-7.3), TCR*β* (H57-597), TCR*γδ* (GL-3), NK1.1 (PK136), NKp46 (29A1.4), IL-7R*α* (A7R34), c-Kit (2B8), Sca-1 (D7), *α*_4_ *β*_7_ (DATK32), Flt3 (A2F10), CD4 (GK1.5), CD45.2 (104), Thy1.2 (53-2.1), ICOS (C398.4A), CD62L (MEL-14), CD69 (H1.2F3), CD44 (IM7), ROR*γ*t (AFKJS-9 or Q31-378), Gata3 (L50-823), human/mouse Ki67 (B56).

Lineage cocktails consisted of

 BM: CD3*ε*, CD5, CD8, CD11c, CD19, TCR*β*, TCR*γδ*, Ter119, Gr1, and NK1.1,
 FL and FS: CD3*ε*, CD11c, CD19, Ter119, Gr1, and NK1.1,
 MLN and LPL: CD3*ε*, CD5, CD8, CD11c, CD19, TCR*β*, TCR*γδ*, Ter119, and Gr1.

All antibodies were purchased from BD Biosciences, eBiosicences, or Biolegend.

### 2.5. Cell Culture

OP9-DL4 stromal cells were plated one day prior to culture experiment in culture medium: OPTIMEM, 10% FCS, *β*-Mercaptoethanol (500 *µ*M, Gibco), penicillin (5 U/mL, Gibco), and streptomycin (5 *µ*g/mL, Gibco). Bone marrow precursors were FACS sorted in 400 *µ*L of culture medium and plated on OP9-DL4 cells at 20 cells per well. Medium was complemented with in-lab produced cytokines (IL-7, c-KitL and Flt3-L). Cells were cultured for 10 days at 37°C and 5% CO_2_ and then harvested and stained for analysis.

### 2.6. *In Vivo* Reconstitution

Ly5.1* Rag2* ^−/−^ *γc* ^−*/*−^ mice were nonlethally irradiated (900 rad) at least 4 hours prior to cell injection. Between 1000 and 2000 Lin^−^  CD4^hi^ IL-7R*α* ^+^ CXCR6^+^ cells from fetal spleens obtained from either E15.5* Cxcr6* ^*Gfp/*+^ or* Cxcr6* ^*Gfp/Gfp*^ embryos were sorted in 100 *µ*L of PBS, and retroorbital injection was performed. Mice were analyzed 4 weeks after injection.

### 2.7. Statistical Analysis

All data were submitted to Student's unpaired bilateral *t*-test. Data were deemed significantly different when ^*∗*^ *p* < 0.05, ^*∗∗*^ *p* < 0.01, or ^*∗∗∗*^ *p* < 0.005.

## 3. Results

### 3.1. CXCR6-Deficient ILC Precursors Are Retained in the Bone Marrow

**Figure 1:**
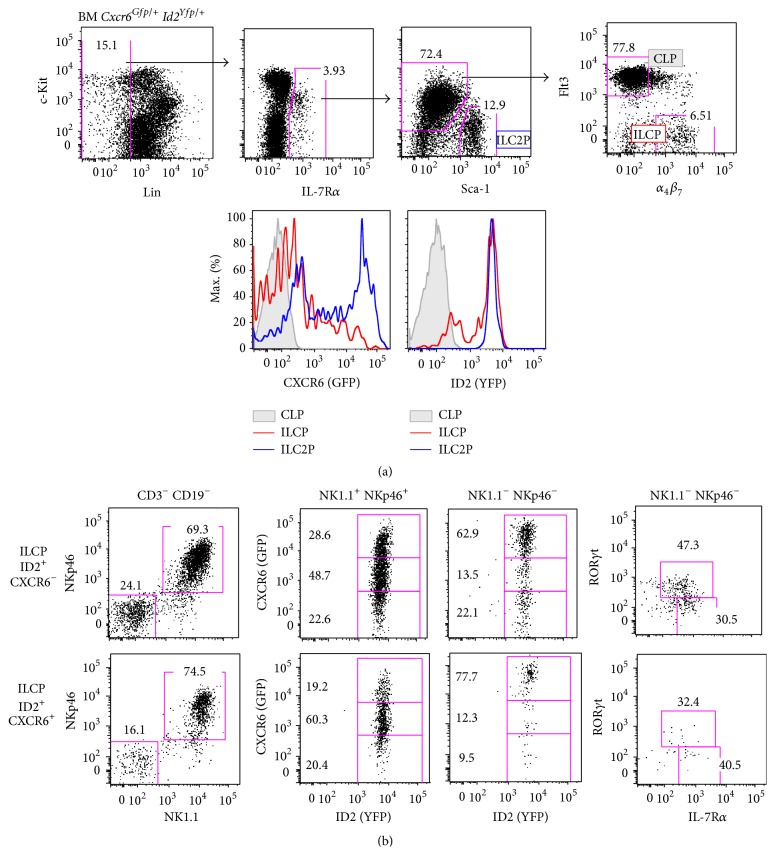
ILCP and ILC2P in the bone marrow heterogeneously express CXCR6. (a) Flow cytometry of lineage depleted (Lin: CD3*ε*, CD5, CD8, CD11c, CD19, TCR*β*, TCR*γδ*, Ter119, Gr1, and NK1.1) adult bone marrow (BM) from* Cxcr6* ^*Gfp/+*^ *Id2* ^*Yfp/+*^ mice. Among Lin^−^ IL-7R*α* ^+^ compartments are defined: CLP (filled gray, c-Kit^lo^  Sca-1^−/lo^ Flt3^+^ *α*_4_ *β*_7_^−^), ILCP (red, c-Kit^lo^  Sca-1^−/lo^ Flt3^−^ *α*_4_ *β*_7_^+^), and ILC2P (blue, c-Kit^−^  Sca-1^hi^). Each compartment is analyzed for CXCR6-GFP and ID2-YFP expression (bottom histograms). (b) Cell culture of BM ILCP, selected as ID2^+^ CXCR6^−^ (upper panels) or ID2^+^ CXCR6^+^ (lower panels). Each population is sorted and cultured at 20 cells per well on OP9-DL4 stromal cells with IL-7, c-KitL, and Flt3L for 10 days and analyzed by flow cytometry. Indicated obtained progenies were analyzed for ID2-YFP, CXCR6-GFP, ROR*γ*t, and IL-7R*α* expression. Results are representative of at least 3 experiments each ((a): *n* > 5), or 2 experiments ((b), at least 4 wells of each condition).

We first characterized the BM lymphoid precursor compartments by crossing and analyzing adult* Id2* ^*Yfp/*+^ *Cxcr6* ^*Gfp/*+^ bone marrow cells [15, 18]. As previously described [14, 19, 20], Lin^−^ IL-7R*α* ^+^ compartment comprises ILC2 precursors (ILC2P) that are Sca-1^hi^  c-Kit^lo^ and lymphoid Sca-1^−/lo^  c-Kit^med^ precursors that are divided into common lymphoid progenitors (CLP; Flt3^+^ *α*_4_ *β*_7_^−^) and *α*_4_ *β*_7_ expressing lymphoid precursors (Flt3^−^ *α*_4_ *β*_7_^+^). The latter expresses ID2 (thus representing ILC precursors or ILCP), as well as ILC2P compartment whereas CLP cells have no expression of ID2. We observe that ILCP and ILC2P comprised both CXCR6^+^ and CXCR6^−^ fractions (Figure 1(a)). CXCR6^hi^ cells are only found in ILC2P fraction (up to 60%).

To confirm the ILC potential of the two different ILCP subsets (CXCR6^+^ and CXCR6^−^), we cultured them on OP9-DL4 stromal cell lines with cytokines to promote cell survival and differentiation (IL-7, c-KitL, and Flt3-L) and analyzed their progeny after 10 days of culture. We confirmed that both CXCR6^+^ and CXCR6^−^ ILCP cells were able to give rise to ILC1 (ID2^+^ NKp46^+^ NK1.1^+^), ILC3 (ID2^+^ NKp46^−^ NK1.1^−^ IL-7R*α* ^+^ ROR*γ*t^+^), and ILC2 (ID2^+^ NKp46^−^ NK1.1^−^ IL-7R*α* ^+^ ROR*γ*t^−^) cells (Figure 1(b)). Interestingly, all ILCs that were obtained expressed variable levels of CXCR6. ILC1 progeny cells were distributed between CXCR6^−^ and CXCR6^hi^ levels, with a majority of CXCR6^int^ cells, regardless of their progenitor (CXCR6^+^ or CXCR6^−^ ILCP), showing that ILC1 could acquire or loose CXCR6 expression. Amongst remaining non-ILC1 cells, all cells are ID2^+^ and are enriched in CXCR6^hi^ cells (up to 77%), but for a smaller fraction that is CXCR6^−^. Because in our previous work, we showed that all ROR*γ*t^+^ cells (which represent between 30% and 50% in NK1.1^−^ NKp46^−^ fraction after culture of ILCP, Figure 1(b)) were comprised of CXCR6^hi^ cells, and the remaining ID2^+^ IL-7R*α* ^+^ ILC2 cells are both CXCR6^hi^ and CXCR6^−^, recapitulating the phenotype of* ex vivo* bone marrow ILC2P.

**Figure 2:**
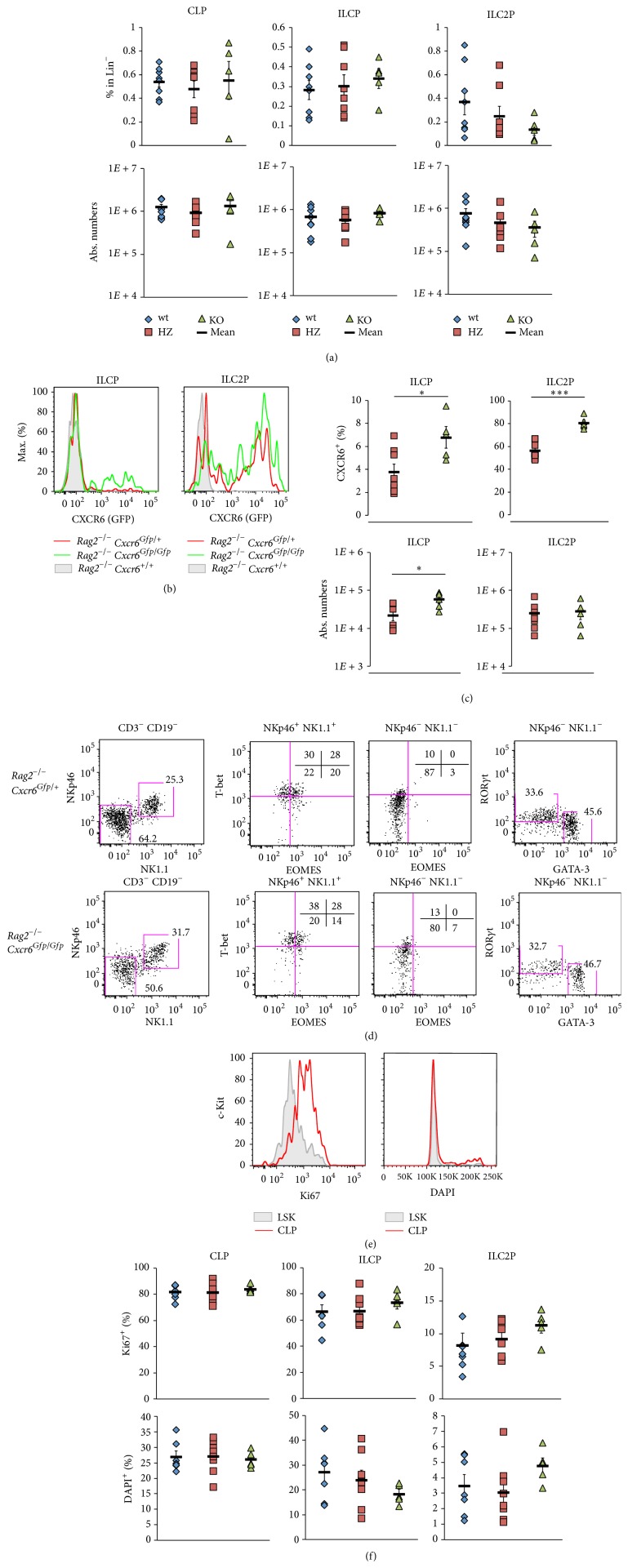
CXCR6-deficient precursors are retained in the bone marrow. (a) Percentages in Lin^−^ (upper panels) and absolute numbers (lower panels) of BM CLP, ILCP, and ILC2P as defined in Figure 1(a) from* Rag2* ^−*/*−^ *Cxcr6* ^+*/*+^ (wt, blue losange),* Cxcr6* ^*Gfp/+*^ (HZ, red square), or* Cxcr6* ^*Gfp/Gfp*^ (KO, green triangle) adult mice. (b) Histograms depicting levels of CXCR6-GFP in ILCP (left panel) or ILC2P (right panel) from* Rag2* ^−*/*−^ *Cxcr6* ^+*/*+^ (filled gray),* Cxcr6* ^*Gfp/+*^ (red line), or* Cxcr6* ^*Gfp/Gfp*^ (green line) bone marrows. (c) Percentages of CXCR6^+^ cells (upper panels) and absolute numbers of (lower panels) BM ILCP (left panels) and ILC2P (right panels) in* Rag2* ^−*/*−^ *Cxcr6* ^*Gfp/+*^ (HZ, red square) or* Cxcr6* ^*Gfp/Gfp*^ (KO, green triangle) adult mice. (d) Cell culture of BM ILCP from* Rag2* ^−*/*−^ *Cxcr6* ^*Gfp/+*^ (upper panels) or* Cxcr6* ^*Gfp/Gfp*^ (lower panels) adult mice. Each population is sorted and cultured at 20 cells per well on OP9-DL4 stromal cells with IL-7, c-KitL, and Flt3-L for 7 days and analyzed by flow cytometry. Indicated obtained progenies were analyzed for T-bet, EOMES, ROR*γ*t, and GATA-3 expression. (e) Histograms of BM LSK cells (filled gray, selected as Lin^−^ IL-7R*α* ^−^  c-Kit^hi^  Sca-1^+^) and CLP (red line) for Ki67 expression and DAPI levels. (f) Percentages of Ki67^+^ cells (upper panels) and DAPI^+^ cells (lower panels) among BM CLP, ILCP, and ILC2P in* Rag2* ^−*/*−^ *Cxcr6* ^*+/+*^ (wt, blue losange),* Cxcr6* ^*Gfp/+*^ (HZ, red square), or* Cxcr6* ^*Gfp/Gfp*^ (KO, green triangle) adult mice. Data are representative of at least 3 experiments ((b), (e): *n* > 5), or are from one experiment ((d), at least 4 wells of each conditions), or are from 3 pooled experiments ((a), (c), (f), wt: *n* = 8, HZ: *n* = 8, KO: *n* = 5). In ((a), (c), (f)), each dot represents a single mouse. Statistical data are displayed with mean and SEM (Student's unpaired bilateral test, ^*∗*^ *p* < 0.05; ^*∗∗*^ *p* < 0.01; ^*∗∗∗*^ *p* < 0.005).

We analyzed then the contribution of CXCR6 to different lymphoid precursor compartments, using* Rag2* ^−/−^ *Cxcr6* ^+*/*+^ (wt),* Cxcr6* ^*Gfp/*+^ (HZ), or* Cxcr6* ^*Gfp/Gfp*^ (KO) mice. As expected, CLP compartment that does not express CXCR6 was not affected by* Cxcr6* deletion either in frequency or in numbers. No difference was observed for total numbers of ILCP and ILC2P in CXCR6-deficient bone marrow (Figure 2(a)). Levels of CXCR6-GFP between HZ and KO mice were not different (Figure 2(b)). However, when looking at the enrichment in CXCR6 expressing cells, a significant increase of CXCR6^+^ precursors is detected in both ILCP and ILC2P subsets (Figure 2(c)). ILCP from both HZ and KO mice were able to give rise to cNK/ILC1 cells (NK1.1^+^ NKp46^+^ T-bet^+^ EOMES^+/−^), ILC2 (NK1.1^−^ NKp46^−^ GATA-3^+^ ROR*γ*t^−^), and ILC3 (NK1.1^−^ NKp46^−^ GATA-3^−^ ROR*γ*t^+^), showing that their differentiation potential was not affected by CXCR6-deficiency (Figure 2(d)). This increase in CXCR6^+^ precursors was not due to a particular proliferative behavior of bone marrow precursors in CXCR6-deficient conditions. The lymphoid precursor subsets display similar frequency of both Ki67^+^ and DAPI^+^ cells in CXCR6-deficient and competent bone marrow (Figures 2(e) and 2(f)).

Overall, our results show that, in CXCR6-deficient mice, a fraction of ILC precursors that still expresses GFP but no functional CXCR6 protein under the control of* Cxcr6 *promoter is retained in the bone marrow. This confinement to the bone marrow concerns both ILCP and committed ILC2P and indicates that CXCR6 contributes to cell egress of all ILC subsets from the bone marrow.

### 3.2. CXCR6 Contributes to Fetal ILC3 and ILC3 Precursor Circulation

**Figure 3:**
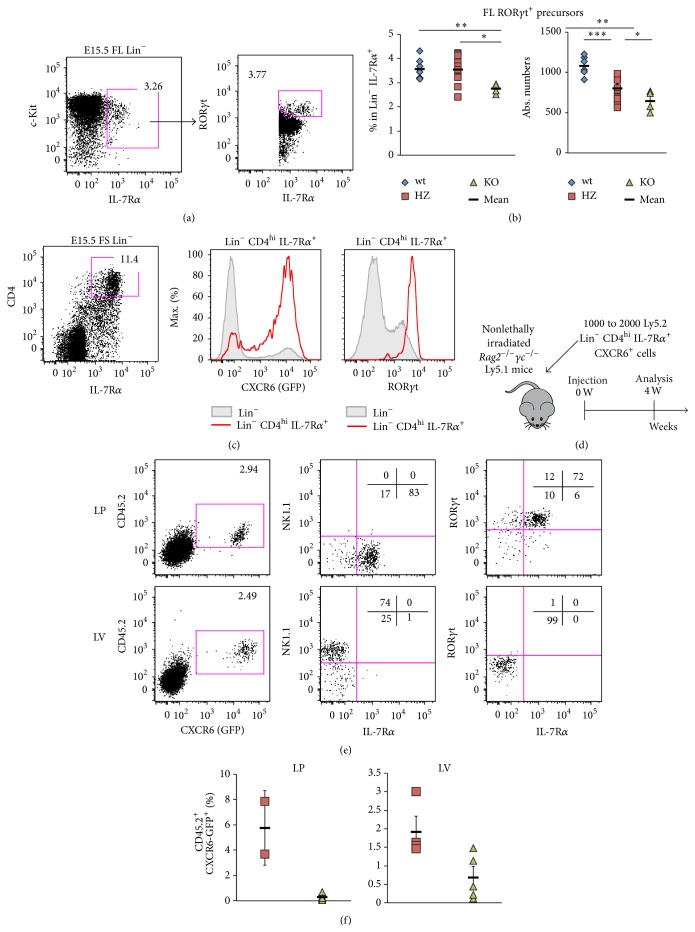
CXCR6 contributes to fetal ILC3 and ILC3 precursor circulation. (a) Flow cytometry of lineage depleted (Lin: CD3*ε*, CD11c, CD19, Ter119, Gr1, NK1.1) E15.5 fetal liver (FL). ILC3 precursors are defined as Lin^−^  IL-7R*α* ^+^  c-Kit^med^ ROR*γ*t^+^. (b) Percentages of Lin^−^ IL-7R*α* ^+^  c-Kit^med^ (left panel) and absolute numbers (right panel) of FL ILC3 precursors as defined in Figure 2(a) in FL from* Cxcr6* ^*+/+*^ (wt, blue losange),* Cxcr6* ^*Gfp/+*^ (HZ, red square) or* Cxcr6* ^*Gfp/Gfp*^ (KO, green triangle) E15.5 embryos. (c) Flow cytometry of Lin^−^ compartment fetal spleen (FS) from* Cxcr6* ^*Gfp/+*^ E15.5 embryos. Lin^−^  CD4^hi^ IL-7R*α* ^+^ cells (red) and total Lin^−^ cells (filled gray) are analyzed for CXCR6-GFP (left histogram) and ROR*γ*t (right histogram). (d) Scheme depicting injection and reconstitution experiment. (e) Analysis of reconstitution experiment. 1000 to 2000 Ly5.2 Lin^−^  CD4^hi^ IL-7R*α* ^+^ CXCR6^+^ cells were injected in nonlethally irradiated* Rag2* ^−*/*−^ *γc* ^−*/*−^ Ly5.1 mice. Recipients were killed 4 weeks after injection and intestinal lamina propria (LP, upper panels) and liver (LV, lower panels) were analyzed by flow cytometry. (f) Percentages of reconstitution experiment as explained in Figures 2(d) and 2(e) using* Cxcr6* ^*Gfp/*+^ (HZ, red squares) or* Cxcr6* ^*Gfp/Gfp*^ (KO) E15.5 embryos in intestinal LP (left panel) or LV (right panel). Results are representative of at least 3 experiments each ((a), (c): *n* > 5), or are from 3 pooled experiments ((b), wt: *n* = 6, HZ: *n* = 10, KO: *n* = 4), or are from at least 2 pooled experiments ((e), (f), LP HZ: *n* = 2, LP KO: *n* = 5, LV HZ: *n* = 4, LV KO: *n* = 5). In ((b), (f)), each dot represents a single mouse. Statistical data are displayed with mean and SEM (Student's unpaired bilateral test, ^*∗*^ *p* < 0.05; ^*∗∗*^ *p* < 0.01).

Our previous work has shown that fetal ILC3 precursors were found during embryonic development in the fetal liver (FL) at embryonic day E15.5, among CXCR6 expressing progenitors [14]. Similarly, to address the contribution of CXCR6 to ILC3 circulation during embryonic development, we analyzed E15.5 FL for the ILC3 precursor compartment, defined as Lin^−^ c-Kit^+^ IL-7R*α* ^+^ ROR*γ*t^+^ (Figure 3(a)) in* Cxcr6* ^+*/*+^ (wt),* Cxcr6* ^*Gfp/*+^ (HZ), or* Cxcr6* ^*Gfp/Gfp*^ (KO) E15.5 FL (Figure 3(b)). We show that frequencies of ILC3 precursors and absolute numbers were significantly reduced, though not totally absent, in KO embryos compared to both HZ and wt embryos (Figure 3(b)).

We next addressed directly the circulation capacities of such CXCR6 expressing ILC3 precursors during adult life. Fetal spleen (FS) Lin^−^  CD4^hi^ IL-7R*α* ^+^ cells are highly enriched in CXCR6 expressing cells and are almost all ROR*γ*t^+^ (Figure 3(c)). Among those cells, we purified the CXCR6^+^ fraction from either* Cxcr6* ^*Gfp/*+^ or* Cxcr6* ^*Gfp/Gfp*^ Ly5.2 embryos and injected them into nonlethally irradiated* Rag2* ^−*/*−^ *γc* ^−*/*−^ Ly5.1 mice. Reconstitution for ILC1 and ILC3 compartments in the intestinal lamina propria (LP) and liver (LV) was analyzed 4 weeks after injection (Figure 3(d)). Among donor CD45.2^+^ cells, LP is mainly composed of NK1.1^−^ IL-7R*α* ^+^ ROR*γ*t^+^ ILC3 cells, whereas LV mainly reconstitutes NK1.1^+^ IL-7R*α* ^−^ ILC1 cells (Figure 3(e)). Interestingly, all reconstituting cells expressed high levels of CXCR6. We clearly observe that CXCR6-deficient progenitor cells fail to reconstitute peripheral compartments, as compared to* Cxcr6* ^*Gfp/*+^ injected ILC3 precursors (Figure 3(f)).

In conclusion, CXCR6 highly contributes to fetal ILC3 precursors and ILC3 circulation, especially towards the intestine and the liver.

### 3.3. Intestinal ILC Compartments Have Heterogeneous Expression of CXCR6 and Egress Markers

**Figure 4:**
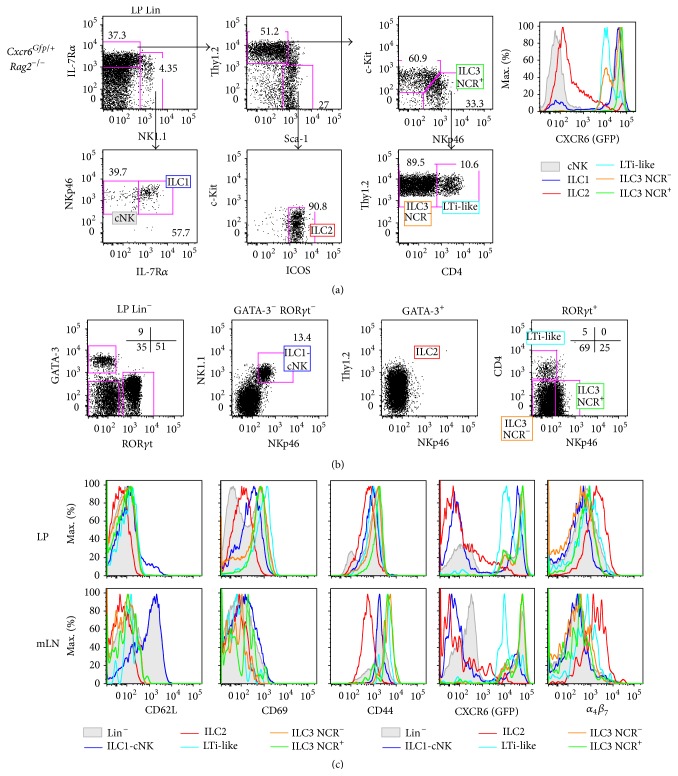
Intestinal ILC compartments have heterogeneous expression of CXCR6 and egress markers. (a) Flow cytometry of Lin^−^ (Lin: CD3*ε*, CD5, CD8, CD11c, CD19, TCR*β*, TCR*γδ*, Ter119, and Gr1) adult intestinal LP from* Rag2* ^−*/*−^ *Cxcr6* ^*Gfp/*+^ mice. ILC subsets are defined as cNK (filled gray, Lin^−^  NK1.1^+^  NKp46^+^ IL-7R*α* ^−^), ILC1 (blue, Lin^−^ NK1.1^+^ NKp46^+^ IL-7R*α* ^−^), ILC2 (red, Lin^−^  NK1.1^−^  Thy1.2^−/lo^  Sca-1^hi^  c-Kit^−^  ICOS^hi^), ILC3 NCR^+^ (green, Lin^−^ NK1.1^−^  Thy1.2^hi^  Sca-1^−/lo^  c-Kit^−^ NKp46^+^), ILC3 NCR^−^ (orange, Lin^−^ NK1.1^−^  Thy1.2^hi^  Sca-1^−/lo^  c-Kit^med^ NKp46^+^ CD4^−^), and LTi-like (light blue, Lin^−^ NK1.1^−^  Thy1.2^hi^  Sca-1^−/lo^  c-Kit^med^ NKp46^+^ CD4^+^). Each compartment is analyzed for CXCR6-GFP expression. (b) Flow cytometry of Lin^−^ adult intestinal LP from* Rag2* ^−*/*−^ *Cxcr6* ^*Gfp/*+^ mice. ILC subsets are defined as ILC2 (red, Lin^−^ GATA-3^+^ ROR*γ*t^−^), ILC3 NCR^+^ (green, Lin^−^ GATA-3^−^ ROR*γ*t^+^ NKp46^+^ CD4^−^), ILC3 NCR^−^ (orange, Lin^−^ GATA-3^−^ ROR*γ*t^+^ NKp46^−^ CD4^−^), LTi-like (light blue, Lin^−^ GATA-3^−^ ROR*γ*t^+^ NKp46^−^ CD4^+^), and ILC1-cNK (blue, Lin^−^ GATA-3^−^ ROR*γ*t^−^ NKp46^+^ NK1.1^+^). (c) Histograms depicting levels of CD62L, CD69, CD44, CXCR6-GFP, and *α*_4_ *β*_7_ in Lin^−^ (filled gray) or ILC subsets (as defined in Figure 3(b)) in adult LP (upper panels) or mesenteric lymph nodes (mLN, lower panels) from* Rag2* ^−*/*−^ *Cxcr6* ^*Gfp/*+^ mice. Results are representative of at least 3 experiments each ((a), (b), (c): *n* > 5).

CXCR6 is heterogeneously expressed by ILC subtypes. By analyzing* Rag2* ^−*/*−^ *Cxcr6* ^*Gfp/*+^ intestinal LP, we show that ILC1 are all CXCR6^hi^ and the diverse ILC3 subsets express different levels of CXCR6, with LTi-like cells expressing lower levels than NCR^+^ and NCR^−^ ILC3 subsets. In contrast, cNK are all CXCR6^−^, and intestinal ILC2 subsets were separated into a large CXCR6^−^ and a smaller CXCR6^+^ fraction (Figure 4(a)).

We further analyzed expression of egress or retention markers such as CD62L, CD69, and CD44, as well as CXCR6 and integrin *α*_4_ *β*_7_ in intestinal LP and also mesenteric lymph nodes (mLN) ILC populations (Figures 4(b) and 4(c)). All ILC subsets expressed CD44 in all organs. Most of the CD62L^+^ mLN cells belong to the ILC1-cNK subset. In the LP, these cells largely become CD62L^−^ as their ILC2 and ILC3 intestinal counterparts. Only intestinal ILC2 subsets were CD69^lo^, whereas intestinal ILC1-cNK and all ILC3 subsets were CD69^+^, suggesting less retention of ILC2 cells in the tissue [21, 22]. In contrast, all mLN ILC subsets were CD69^−^. CXCR6-GFP expression pattern in mLN ILC subsets is similar to what could be observed in LP ILC subsets (Figure 4(a)). Finally, integrin *α*_4_ *β*_7_ was only detected in ILC2 subset but not in other ILC subsets in both LP and mLN (Figure 4(c)).

In conclusion, only intestinal ILC1 and ILC3 subsets that express high levels of both CXCR6 and CD69 are more likely to be tissue resident ILC populations.

### 3.4. Normal Intestinal ILC1 and ILC3 Compartments in CXCR6-Deficient Mice Are Compensated by Higher Proliferative Capacities of Respective ILC Compartments and of an* In Situ* Progenitor Cell

**Figure 5:**
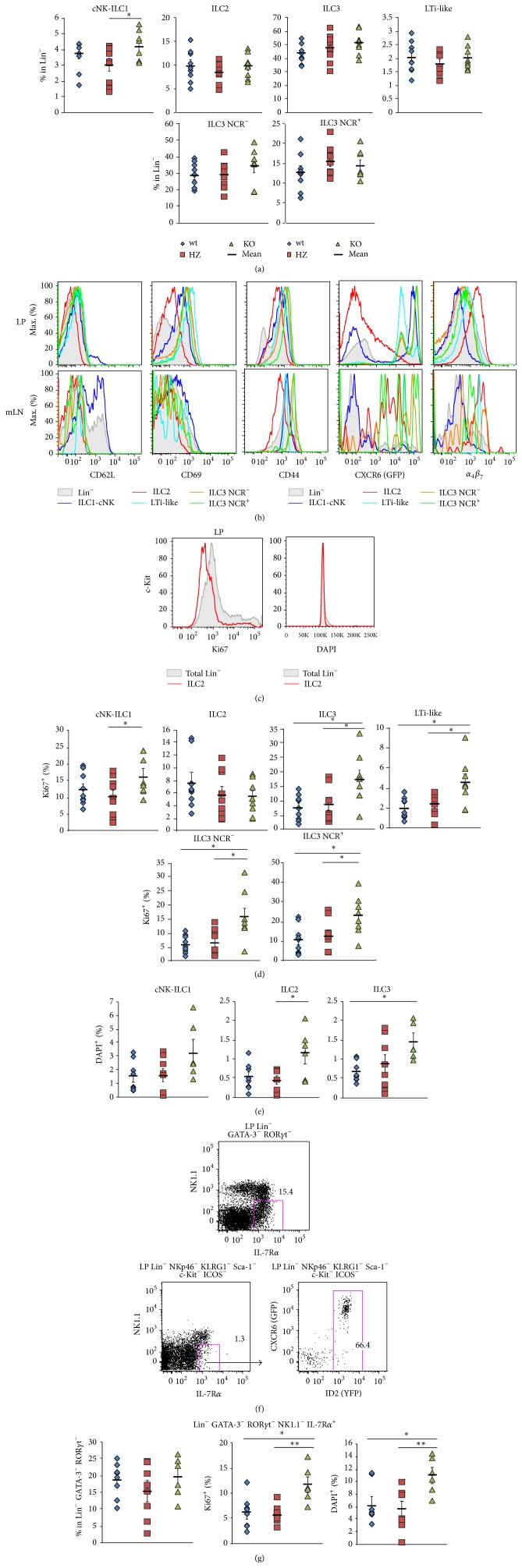
Normal intestinal ILC compartments in CXCR6-deficient mice are compensated by higher proliferative capacities of* in situ* progenitor cell. (a) Percentages of ILC subsets in Lin^−^ (as defined in Figure 3(b)) in adult LP from* Rag2* ^−*/*−^ *Cxcr6* ^*+/+*^ (wt, blue losange),* Cxcr6* ^*Gfp/+*^ (HZ, red square), or* Cxcr6* ^*Gfp/Gfp*^ (KO, green triangle) adult mice. (b) Histograms depicting levels of CD62L, CD69, CD44, CXCR6-GFP, and *α*_4_ *β*_7_ in Lin^−^ (filled gray) or ILC subsets (as defined in Figure 3(b)) in adult LP (upper panels) or mesenteric lymph nodes (mLN, lower panels) from* Rag2* ^−*/*−^ *Cxcr6* ^*Gfp/Gfp*^ mice. (c) Histograms of total LP Lin^−^ cells (filled gray) or ILC2 cells (red line) for Ki67 expression and DAPI levels. ((d), (e)) Percentages of Ki67^+^ cells (d) and DAPI^+^ cells (e) among ILC subsets (as defined in Figure 4(b)) in LP from* Rag2* ^−*/*−^ *Cxcr6* ^*+/+*^ (wt, blue losange),* Cxcr6* ^*Gfp/+*^ (HZ, red square), or* Cxcr6* ^*Gfp/Gfp*^ (KO, green triangle) adult mice. (f) Flow cytometry of LP* in situ* ILCP (upper panel) and CXCR6-GFP and ID2-YFP expression of LP* in situ* ILCP (lower panels) from* Cxcr6* ^*Gfp/+*^ *Id2* ^*Yfp/+*^ mice. (g) Percentages of IL-7R*α* ^+^ precursors in Lin^−^ (left), Ki67^+^ (middle), and DAPI^+^ (right) cells among IL-7R*α* ^+^ precursors in LP from* Rag2* ^−*/*−^ *Cxcr6* ^*+/+*^ (wt, blue losange),* Cxcr6* ^*Gfp/+*^ (HZ, red square), or* Cxcr6* ^*Gfp/Gfp*^ (KO, green triangle) adult mice. Results are from 3 pooled experiments ((a), (c), (d): wt: *n* = 9, HZ: *n* = 9, KO: *n* = 8) or from 2 pooled experiments ((e), (f), GF, wt: *n* = 8, HZ: *n* = 8, KO: *n* = 6) or are representative of 3 experiments (b). In ((a), (d), (e), (g)), each dot represents a single mouse. Statistical data are displayed with mean and SEM (Student's unpaired bilateral test, ^*∗*^ *p* < 0.05; ^*∗∗*^ *p* < 0.01).

We confirmed that, in CXCR6-deficient mice, there was no impact on the repartition of the different ILC subsets at homeostasis, except for cNK-ILC1 that are significantly (but slightly only) increased in CXCR6-deficient LP (Figure 5(a)), in line with previous publications [16]. Overall, the balance of intestinal ILC subsets was not majorly affected, as we observed similar repartitions between wt, HZ, and KO mice. Intestinal ILC are mainly composed of 45% to 50% of ILC3 (with a minority of 2% of LTi-like cells, roughly 15% of ILC3 NCR^+^ cells, and mainly ILC3 NCR^−^ cells), then a subsequent part of ILC2 (10%), and a minority of cNK-ILC1 cells (3% to 4%) (Figure 5(a)).

Similar to HZ mice, we tested CD69, CD62L, CD44, CXCR6-GFP, and integrin *α*_4_ *β*_7_ expression among ILC subsets in LP and mLN from KO mice. We show that the expression pattern of those markers was not significantly perturbed by CXCR6-deficiency (Figure 5(b)).

Then, we tested whether each of the ILC subsets was similarly distributed between the different stages of proliferation (Figure 5(c)). We defined that there was an important increase (up to two fold increase) in Ki67^+^ cells amongst cNK-ILC1 and all ILC3 subsets between HZ or wt control and CXCR6-deficient conditions (Figures 5(c) and 5(d)). In contrast, the Ki67^+^ frequency of ILC2 cells remained unchanged. Additionally, DAPI staining showed that all ILC subsets were enriched in cells that were in S-G2-M stages (i.e., DAPI^+^) in the CXCR6-deficient condition (Figure 5(e)). These results show that intestinal ILC1 and ILC3 compartments, and to a certain extent ILC2, were seeded with dividing DAPI^+^ and active recently divided Ki67^+^ cells, after CXCR6 ablation.

We analyzed the putative* in situ *intestinal ILC precursor fraction that is isolated as Lin^−^ NKp46^−^ NK1.1^−^ GATA3^−^ ROR*γ*t^−^ IL-7R*α* ^+^ cells (Figure 5(f)). This fraction, gated using surrogate markers as described to identify ILC subsets in* Cxcr6* ^*Gfp/*+^ *Id2* ^*Yfp/*+^ mice (as in Figure 4(a)), expresses CXCR6 and ID2 (Figure 5(f)). No difference in the frequency of those cells is observed, but they were significantly enriched in both Ki67^+^ (active G1-S-G2 cells) and DAPI^+^ (dividing S-G2-M cells) in CXCR6-deficient intestines (Figure 5(g)). This result determines that this compartment is highly active in the CXCR6-deficient mice and contributes to proliferation, thus differentiation to seed the respective mature ILC compartments.

In conclusion, these results show that* in situ* intestinal ILC precursor cells compensate a defect in homing to the intestine by homeostatically proliferating and result in a proper balance of diverse ILC compartments.

## 4. Discussion

ILCs share numerous characteristics with the T-helper cell subsets and we considered that they might have similarities in their trafficking features even if they do not have to encounter antigen. For naïve T lymphocytes, it was demonstrated that the intestinal homing marker *α*_4_ *β*_7_ is upregulated after their activation in the mLN [23]. For the ILC lineage, this intestinal homing marker is already expressed by their precursors in the bone marrow. Moreover, CXCR6 was also shown to be expressed by the most mature fraction of *α*_4_ *β*_7_^+^ medular ILC precursors. Hence, CXCR6 may represent one of the chemokine receptors important for the egress and homing of ILC precursors. We decided to study the role of CXCR6 by comparing diverse features of ILC populations between* Cxcr6* ^*Gfp/*+^ and* Cxcr6* ^*Gfp/Gfp*^ mice in steady state conditions. We crossed our mouse models to obtain* Rag2* ^−*/*−^ *Cxcr6* ^*Gfp/*+^ and* Rag2* ^−*/*−^ *Cxcr6* ^*Gfp/Gfp*^ mice since most studies of ILC function count on* Rag2* ^−*/*−^ mice to avoid an important contamination of T cells.

The determination of CXCR6 expression fractions among the ID2^+^ ILC precursors was performed using* Cxcr6* ^*Gfp/*+^ *Id2* ^*Yfp/*+^ double reporter mice and showed that the ILCP (Lin^−^ IL-7R*α* ^+^  Sca-1^−/lo^  c-Kit^med^  Flt3^−^ *α*_4_ *β*_7_^+^) and ILC2P (Lin^−^ IL-7R*α* ^+^  Sca-1^hi^  c-Kit^lo^) subsets are inversely enriched into CXCR6^+^ precursors, with few CXCR6^+^ cells among ILCP and a substantial fraction of CXCR6^+^ cells among ILC2P.

Here, we showed that deficiency of the chemokine receptor CXCR6 leads to increased number of all bone marrow CXCR6-GFP ILC precursors in homeostatic conditions. This observation suggested that CXCR6 could either regulate the proliferation of ILCP/ILC2P or could be implicated in the egress of those precursors to the peripheral organs. Hence, we analyzed the proliferative status of the bone marrow progenitors and demonstrated that CXCR6 is not implicated in the proliferation of these progenitor pools but participates in their specific egress. Thus, we further analyzed how the CXCR6 expression affects the steady-state number of the different ILC subsets* in vivo* in the periphery. Intestinal lamina propria was analyzed for the repartition of the different ILC compartments in absence of CXCR6. Despite the retention of ILC precursors in the bone marrow, we observed comparable frequencies for mature ILC2 and ILC3 intestinal subsets between* Rag2* ^−*/*−^ *Cxcr6* ^*Gfp/*+^ and* Rag2* ^−*/*−^ *Cxcr6* ^*Gfp/Gfp*^ littermates. Hence, under homeostatic conditions, the deficiency in CXCR6 does not affect the distribution of ILC2 and ILC3 subsets in the intestine as also recently shown using CXCR6-deficient mice [16].

The absence of difference in ILC frequencies, at steady state, suggested that CXCR6 was not required for the development or tissue homing of ILC [16]. However, since we observed that the ILC precursors are partially retained in the bone marrow and previously showed their homing to the periphery for final differentiation towards the ILC3 lineage [14], we suspected CXCR6 as a specific chemokine receptor for initiating the ILCP egress and homing. Then, we decided to check for the seeding and frequency of this progenitor compartment in the periphery. No decrease in the frequency of this progenitor was observed. By analyzing its homeostatic* in situ* proliferation, we proved that increased proliferation of this progenitor in the intestine is one explanation for the normal distribution of ILC subsets in CXCR6-deficient mice despite the partial bone marrow retention.

We also used the combination of Ki67/DAPI to examine the mature ILC subsets and showed that, in CXCR6-deficient mice, all ILC subsets are more frequent in proliferation or in active state in the intestine. ILC subsets in the intestinal LP were already shown to be more activated than ILCs in other tissues, probably because of their constant exposure to varied environmental signals [24]. The analyses of intestinal Ki67^+^ ILC subsets demonstrated that both recent proliferative ILC1 and ILC3 subsets stay in the intestine in CXCR6 deficient mice whereas ILC2 tends to leave the organ since no increase of Ki67^+^ ILC2 cells was detected in CXCR6-deficient intestines. This result is consistent with the observation that intestinal ILC2 subsets express lower levels of the resident marker CD69 and lower levels of CXCR6 than ILC3 subsets. It has been previously shown that, in the intestinal lamina propria, the diverse ILC3 subsets express CXCR6 at different levels with higher levels for the NCR^+^ ILC3 subset [25]. We found similar results in* Rag2* ^−*/*−^ *Cxcr6* ^*Gfp/*+^ intestines where the GFP expression is the highest in NCR^+^ ILC3 and in most NCR^−^ ILC3 cells and lower in both CD4^+^ LTi-like ILC3 and a small NCR^−^ ILC3 subset. In infectious conditions, a specific reduction of NCR^+^ ILC3 subset was shown in CXCR6-deficient mice [25]. Indeed,* Citrobacter rodentium* infection challenges have recently defined that CXCR6 may highly contribute to the localization of NCR^+^ ILC3 subsets and its capacity to eliminate the bacterial load. The CXCR6 ligand (CXCL16) is expressed in the intestine by a specific population of CX3CR1^+^ DC cells [26] and was reported to be crucial for NCR^+^ ILC3 stimulation and production of IL-22 [25]. However, the regulation of ILC function could be different depending on the ILC subset since numerous types of cells, such as epithelial cells, monocytes, and macrophages, also express CXCL16.

CXCR6 is also important for circulation of ILC3 and ILC3P in the embryo since we observed a significant decrease of ROR*γ*t^+^ cells in the fetal liver of CXCR6-deficient mice. We already assessed the effect of CXCR6 deficiency in ILC3 fetal differentiation and demonstrated that CXCR6 deficiency does not affect ILC3 differentiation during embryogenesis [14]. Since* Cxcr6* ^*Gfp/Gfp*^ mice possess normal development of Peyer's patches and lymph nodes [14, 25], the homing of ILC3P is not impaired. However, circulation of these progenitors could be affected. Indeed, ROR*γ*t^+^ cells are rare in the fetal liver and could be considered as partly derived from the circulation. Hence, we suspected an important role of CXCR6 for the recirculation of ILC3 precursors and ILC3 subsets. We demonstrated by reconstitution assays that, in absence of CXCR6 expression, ILC3P are unable to correctly reach the peripheral organs contrary to CXCR6-competent precursors. Reconstitutions were performed using i.v. injection suggesting that recirculation via the blood circuit is altered in absence of CXCR6 expression.

A study using Kaede transgenic mice demonstrated a constitutive trafficking of ILC from the gut to the mLN where LTi like-cells ILC3 migration was dependent on CCR7 in contrary to other ILC subsets [27]. The predominance of some ILC3 over other ILC subsets was already described in LN and it was observed that, in lymphopenic mLN, the ILC frequencies are altered [27], so we decided to look for ILC homing markers expression in both CXCR6 competent and deficient mLN but not for ILC respective repartition. We found that markers are maintained after CXCR6 loss and determined that ILCs were mainly trafficking between the intestine and mLN since they were all CD44^+^ CD69^−^ in mLN. All ILC subsets except some ILC1 were clearly negative for CD62L, showing no preferential circulation through lymphoid organs.

In conclusion, we provide here new data on the role of CXCR6 during circulation of ILC progenitors and ILC populations at steady state. ILC precursors that are deficient for CXCR6 are retained in the bone marrow and only few of them could reach the blood and colonize peripheral lymphoid organs to continue their maturation. However, we demonstrated that homeostatic proliferation is increased in these CXCR6 deficient animals to compensate for the reduction of seeding precursors resulting in normal ILC subset distribution. Future studies concerning the function of this receptor during inflammation and infection should now take into account the importance of this receptor on ILC recirculation capacities.
